# Identifying Insomnia From Social Media Posts: Psycholinguistic Analyses of User Tweets

**DOI:** 10.2196/27613

**Published:** 2021-12-09

**Authors:** Ahmed Shahriar Sakib, Md Saddam Hossain Mukta, Fariha Rowshan Huda, A K M Najmul Islam, Tohedul Islam, Mohammed Eunus Ali

**Affiliations:** 1 American International University-Bangladesh Dhaka Bangladesh; 2 United International University Dhaka Bangladesh; 3 LUT University Lappeenranta Finland; 4 Bangladesh University of Engineering and Technology Dhaka Bangladesh

**Keywords:** insomnia, Twitter, word embedding, Big 5 personality traits, classification, social media, prediction model, psycholinguistics

## Abstract

**Background:**

Many people suffer from insomnia, a sleep disorder characterized by difficulty falling and staying asleep during the night. As social media have become a ubiquitous platform to share users’ thoughts, opinions, activities, and preferences with their friends and acquaintances, the shared content across these platforms can be used to diagnose different health problems, including insomnia. Only a few recent studies have examined the prediction of insomnia from Twitter data, and we found research gaps in predicting insomnia from word usage patterns and correlations between users’ insomnia and their Big 5 personality traits as derived from social media interactions.

**Objective:**

The purpose of this study is to build an insomnia prediction model from users’ psycholinguistic patterns, including the elements of word usage, semantics, and their Big 5 personality traits as derived from tweets.

**Methods:**

In this paper, we exploited both psycholinguistic and personality traits derived from tweets to identify insomnia patients. First, we built psycholinguistic profiles of the users from their word choices and the semantic relationships between the words of their tweets. We then determined the relationship between a users’ personality traits and insomnia. Finally, we built a double-weighted ensemble classification model to predict insomnia from both psycholinguistic and personality traits as derived from user tweets.

**Results:**

Our classification model showed strong prediction potential (78.8%) to predict insomnia from tweets. As insomniacs are generally ill-tempered and feel more stress and mental exhaustion, we observed significant correlations of certain word usage patterns among them. They tend to use negative words (eg, “no,” “not,” “never”). Some people frequently use swear words (eg, “damn,” “piss,” “fuck”) with strong temperament. They also use anxious (eg, “worried,” “fearful,” “nervous”) and sad (eg, “crying,” “grief,” “sad”) words in their tweets. We also found that the users with high neuroticism and conscientiousness scores for the Big 5 personality traits likely have strong correlations with insomnia. Additionally, we observed that users with high conscientiousness scores have strong correlations with insomnia patterns, while negative correlation between extraversion and insomnia was also found.

**Conclusions:**

Our model can help predict insomnia from users’ social media interactions. Thus, incorporating our model into a software system can help family members detect insomnia problems in individuals before they become worse. The software system can also help doctors to diagnose possible insomnia in patients.

## Introduction

### Background

Insomnia, a type of sleep disorder, is the inability to fall asleep or stay asleep at night. It is one of the most prevalent mental health symptoms globally [1]. One study [2] suggests that approximately 30% of adults worldwide exhibit insomnia symptoms, like difficulty initiating and maintaining sleep and waking up too early. People with insomnia might also experience other problems, such as depression, anxiety, and excessive alcohol consumption [3].

With the unprecedented growth of smartphone and internet technologies, social media has now become a ubiquitous platform that reflects users’ daily activities, preferences, and beliefs. These social media platforms have become a means to share health information [4-9] for many users. For example, Paul et al [10] have stated that Twitter has become a common place to discuss a wide range of health information, including insomnia and other mental health conditions such as depression, stress, and anxiety. Several other studies [10-12] also report that Twitter is used as a platform to share symptoms [11], seek help, and exchange advice [12].

As insomnia is a mental health disorder, the illness might have a strong connection with human personality attributes. In fact, prior studies [13-15] suggest that insomnia has links with certain personality traits. Therefore, in this study, we attempted to derive personality traits from users’ social media interactions and use these traits along with users’ word usage patterns to predict insomnia. To the best of our knowledge, our study is the first to investigate from social media interactions whether personality traits have an association with insomnia. Predicting insomnia by analyzing users’ tweets has a number of real-life applications. For example, friends and parents can identify problems in their loved ones, while health care providers can use the system to diagnose insomnia and can build an automated early warning system.

### Insomnia and the Big 5 Personality Traits

Many adults experience short-term (acute) insomnia, which lasts for days or weeks. Acute insomnia is common and often brought on by situations such as stress at work, family pressures, or a traumatic event. Some adults have long-term (chronic) insomnia that lasts for several months or years [16]. In most cases, chronic insomnia may be a side effect of other problems [16]. Insomnia not only reduces individuals’ energy levels but also degrades their health, work performance, and quality of life. There are several causes of insomnia [16-18], including mental health disorders, such as posttraumatic stress disorder. Antidepressants, asthma medications, and blood pressure medications can also lead to sleep disorders. Medical conditions such as chronic pain, cancer, diabetes, heart disease, asthma, gastroesophageal reflux disease, overactive thyroid, Parkinson disease, and Alzheimer disease can also cause insomnia. High consumption of caffeine, nicotine, or alcohol may also prevent sleep and lead to insomnia [16].

Personality differentiates individuals in their patterns of thinking, feeling, and behaving [19]. The Big 5 personality scale is a common scale for measuring personality [19,20]. The Big 5 model has 5 different personality traits: openness, conscientiousness, extroversion, agreeableness, and neuroticism. People with a high level of openness have a tendency to reflect on ideas, innovate, and appreciate values. People with high conscientiousness are cautious and meticulous and tend to seek achievement. People with high extroversion have a tendency to seek excitement and show positive emotions. People with high agreeableness tend to be sympathetic, trusted, and merciful to others. Neurotic individuals show negative emotions, such as anxiety, inhibition, anger, and depression. Personality traits are important factors that are associated with disorders [21]. For example, prior research has found that conscientiousness and neuroticism are related to insomnia [22,23]. Personality is also the key factor for dropout and treatment resistance in cognitive behavioral therapy for insomnia [24].

### Social Media and Insomniac Patterns

A few studies have been conducted to predict insomnia by analyzing the content of social media. Michael et al [10] described a method that uses Twitter for public health research. They exploited the Ailment Topic Aspect Model to create structured disease information from tweets that they used for public health metrics. These authors [25] also reported that early detection of disease outbreaks, medication safety, health behaviors, and individual well-being can be investigated through social media data, and applied traditional natural language processing tools to analyze social media content. Rice et al [26] found that young people may be at risk of negative consequences from new technologies and online media. Therefore, social media platforms are important for measuring young people’s mental health states. Andrew et al [27] conducted a study to identify common mental health topics from popular social media platforms and identified common mental health topics such as anxiety, depression, and sleep problems. Jamison-Powell et al [9] completed a study on discussions of insomnia on Twitter. Through an analysis of 18,901 tweets, they discovered that when the word “insomnia” appears in users’ tweets, they are likely to convey strong negative health information. These authors mainly conducted their analysis over 2 different themes: coping with insomnia and describing experiences with insomnia. For the first theme, users share symptoms and coping strategies on Twitter, while for the second theme, users share frustration. However, the authors did not build a prediction model and did not explore the association between users’ personality and insomnia. McIver et al [28] examined how 2 groups of Twitter users—sleep and nonsleep groups—were active on social media. They found that the nonsleep group showed negative sentiment over social media. Suarez et al [29] conducted a study on the real-time streaming of Spanish tweets for insomnia prediction by analyzing 54,432 tweets and built a classifier whenever insomnia phrases appeared. They used term frequency-inverse document frequency to find features of n-grams and then applied different classifiers including support vector machines and k-nearest neighbors.

It is clear that prior studies suffer from the following deficits in research: prior studies largely built classifiers based on explicit insomnia phrases (eg, “insomnia,” “sleepless”) rather than the linguistic cues and relationships between the words, the authors did not build any novel machine learning models, and, to the best of our knowledge, no study predicted insomnia based on users’ personalities from social media interactions. The aim of this paper was thus to address these research gaps.

### Research Objectives

The main objective of this study is to predict insomnia by analyzing users’ psycholinguistic characteristics (eg, word usage patterns) and Big 5 personality traits as derived from their tweets. In this study, we built a rigorous classification model to predict users’ insomnia from their interactions on Twitter. We collected a total of 4,198,683 tweets from 1574 users and classified our training data set into 2 classes: individuals who suffer from insomnia and individuals who do not suffer from insomnia. We then conducted psycholinguistic-based analyses of users’ tweets by using 2 popular tools: Empath [30] and Linguistic Inquiry and Word Count (LIWC) [31]. We also analyzed users’ tweets by using the bidirectional encoder representations from transformers (BERT) [32] word embedding technique to investigate semantic relationships between words. We carefully conducted both psycholinguistic- and word embedding–based analyses to find insights into users’ tweets. We then integrated users’ personalities with the psycholinguistic models. Finally, we built double-weighted, ensemble-based classification models by combining psycholinguistic-, word embedding–, and personality-based models.

In summary, our study provides the following contributions: a large insomnia data set consisting of 1574 users from different geographical locations; novel insomnia classification models comprising psycholinguistic, word embedding, and Big 5 personality attributes as derived from users’ tweets; and a novel and rigorous double-weighted, ensemble-based classification model to predict insomnia.

## Methods

In this paper, we performed the 5 following steps to predict insomnia by analyzing users’ word use patterns in tweets: (1) user selection—first, we found a total of 1574 users by using Twitter’s advanced search technique [33] and divided the users into 2 groups, users with sleep disorders and users with no sleep disorder; (2) linguistic analysis—we performed 2 different types of linguistic analyses, psycholinguistic-based analysis and word embedding–based analysis; (3) correlation analysis—we found correlations between users’ psycholinguistic patterns and sleep disorders by using Fisher’s latent Dirichlet allocation (LDA) [34]; (4) model building—we built 3 different machine learning models by using psycholinguistic features, word embedding attributes, and the Big 5 personality traits; and (5) ensemble of the models—finally, we built a double-weighted ensemble model by integrating the previous models. These steps are further detailed in the subsequent subsections.

### Data Collection

We collected tweets from a total of 1574 users. We searched phrases such as “insomnia” and “sleepless” by using Twitter’s advanced search technique. We divided the users into 2 groups: Insomnia Yes and Insomnia No. We then manually annotated the files of the users’ tweets with the Insomnia Yes and Insomnia No labels.

We confirmed that randomly selected users were neither organizational nor celebrity profiles. We manually checked whether Insomnia Yes users disclosed their own sleep disorders. We found users’ tweets and their locations. The following are a few sample tweets shared by insomniac users on Twitter: “I wish insomnia wasn’t a part of my life” and “my insomnia is officially the worst I got into bed at 12, slept for 2 hours, and am still wide awake at 6:30 AM.” These tweets indicated that these users suffered from insomnia. If we found several such tweets (28.60 times on average) in the newsfeed or tweets of a user, we labeled that user as Insomnia Yes.

For Insomnia No users, we randomly selected users who did not have these search phrases in their tweets. We also manually checked whether these users shared any sleeping-related issues, and if they did, we discarded them from the list. After labeling Insomnia Yes or No on the users’ csv files containing the tweets, we removed text such as “insomnia” and “sleepless” from the users’ tweets. By removing these phrases, we created a bias-free data set that contained cues regarding users’ insomnia without explicitly mentioning these phrases. Users who used contractions tend to have higher levels of fluency in their pronunciation, while users who do not use contractions are largely nonnative speakers. People may contract many words every day but mainly focus on the word “not” during contraction [35]. For the above reasons, we kept contracted words in our tweets to indicate a difference between the patterns of writing between native and nonnative speakers. Later, we conducted our full analysis again.

It is important to note that we only focused on users who express their insomnia on Twitter. If they are actually suffering from insomnia, then their word usage pattern could be insightful. We collected users’ tweets, and multiple referees labeled these users after investigating their writing, their geographic locations, and the irregular timestamps of the tweets. In this way, we could be confident that a given user was suffering from insomnia. We emphasized a users’ concern from their tweet content to make decisions about their behavior. It is likely that when users encounter a grave issue, they frequently express their problem to others. Thus, we labeled a user as Insomnia Yes if we found a high frequency (average 28.60) of mentions of the problem. We also found the maximum, minimum, and SD of users’ number of posted tweets regarding the aforementioned problem to be 363, 8, and 24.57, respectively. These numbers revealed that some users suffering from extreme insomnia-related problems continue to tweet at nighttime about their sleeplessness.

There might be another group of users who have insomnia but do not disclose the problem in their tweets. We did not consider those users in our study. We also found a large number of users who were noninsomniacs. We again manually checked the users’ data and tweet times based on geographic location to confirm that these users were noninsomniacs. If we had users who were noninsomniacs but tweeted in different time patterns to regular users, then we discarded their tweets from our study. As the number of insomniac users is lower in comparison to noninsomniac users, we took less data of noninsomniac users to make a balanced data set.

It is important to note that there might have been users who had insomnia but do not disclose it in their tweets. However, we argue that they are not in large numbers, as previous studies show that in social media people reveal their actual behavior without idealization [36] and share private traits [37].

Next, we identified the genders of Twitter users based on various attributes to investigate correlations between users’ gender and insomniac behavior. First, we manually checked profile pictures and biographies to identify gender, but many users do not share their photos in their profiles. Thus, we observed their writing and the replies of other users to determine their gender from third person pronouns (ie, “he,” “she,” “him,” and “her”). If we could not identify the gender from Twitter, we then checked the other social network accounts of that particular user by conducting a manual search based on their names and usernames. For instance, we could identify the genders of Twitter users based on their pictures on their Instagram or Facebook profiles. If all the above methods failed to ascertain the gender, we discarded the user from our list.

We searched for users from those countries that have English as their first language and were either native or nonnative speakers. We collected users’ tweets from a total of 6 different countries: Australia, Canada, Ireland, New Zealand, the USA, and the UK. Table 1 shows the number of users from both groups (Insomnia Yes and Insomnia No) collected from different geographical locations.

Table 2 displays the statistics describing the tweets of our data set.

**Table 1:** Twitter users’ statistics by location.

Countries	Total, n (N=1574)	Insomnia Yes users, n (n=820)	Insomnia No users, n (n=754)
UK	212	108	104
Australia	62	31	31
Canada	334	181	153
USA	919	473	446
New Zealand	32	18	14
Ireland	15	9	6

**Table 2:** Tweet statistics.

Statistic	Insomnia Yes	Insomnia No
Tweets, n	1,998,683	1,810,567
Maximum number of tweets of a user, n	3247	3250
Minimum number of tweets of a user, n	26	26
Average number of tweets of a user, mean (SD)	2437.42 (1035.42)	2401.28 (1156.65)
Maximum word count of a user, n	67,427	65,660
Minimum word count of a user, n	195	191

For raw data preprocessing, we discarded the username and mentions. We kept the retweets of users because the act of retweeting could indicate the personality traits of users. We removed hashtags (eg, “#insomnia,”) and converted them into text (eg, “insomnia”). We also removed URLs and http links because these text data cannot be analyzed by lexical methods. These texts also do not produce any sensible numeric vector for the word embedding method. We did not remove stop words because some word embedding techniques, like BERT, show that prepositions facilitate a better understanding of the context. We removed emojis by using the Python demoji package [38]. For example, we replaced the fire symbol emoji with the word “fire.” We used the LIWC2015 dictionary for removing emoticons according to the suggestion of Seabrook et al [39].

### Model Building

We built the classification models from 3 different techniques: (1) a psycholinguistic-based model (ie, LIWC and Empath), (2) a word embedding–based model (ie, BERT), and (3) a Big 5 personality–based model. First, we describe the performance of predicting Insomnia Yes and Insomnia No for each of the independent models. We then describe the process of building our novel ensemble model from these 3 independent models. Since we used word-embedding techniques for deep learning–based models, we did not need a feature selection method for this classification category. We carefully selected important features for the other 2 approaches because irrelevant features can weaken the accuracy of a model [40].

#### Feature Selection

In our data set, our independent variables consisted of analysis scores from users’ tweets as generated by psycholinguistic tools (ie, LIWC and Empath), word-embedding methods (ie, BERT), and the Big 5 personality traits. Our dependent variable was Insomnia Yes and Insomnia No. As our independent variables were continuous while the dependent variable was categorical, we applied Fisher’ linear discriminant analysis [34] for our feature selection method.

As mentioned earlier, we used 2 different psycholinguistic techniques, LIWC and Empath, to analyze users’ tweets. Initially, we used the LIWC-based approach using LIWC2015, which classifies approximately 90 different features from texts into 7 different categories, where each category contains hundreds of words [41]. These categories include summary language variables (analytical thinking, clout, authenticity, and emotional tone), general descriptor categories (words per sentence, percent of target words captured by the dictionary, etc), standard linguistic dimensions (articles, auxiliary verbs, etc), word categories tapping psychological constructs (affect, cognition, etc), personal concern categories (work, home, leisure activities, etc), informal language markers (assents, fillers, swear words, netspeak), and punctuation categories (periods, commas, etc).

We first considered LIWC scores to be independent variables and Insomnia Yes and Insomnia No as dependent variables. We applied Fisher’s linear discriminant analysis by using SPSS statistical software (IBM Corp) to find the relevant features. Table 3 shows the correlation coefficient between users’ LIWC scores and Insomnia Yes and Insomnia No according to Fisher’s linear discriminant analysis. The predictors with larger (>1.0) scores are better predictors. Therefore, these scores are helpful in deciding which variables have more effect when building the classification models [42].

**Table 3:** Fisher’s correlation coefficient between LIWC^a^ categories and insomnia categories.

LIWC category	Insomnia Yes	Insomnia No
i	48.117	47.838
negate	123.587	123.253
swear	74.714	74.521
health	18.731	18.466
drives	–31.599	–31.479
focuspresent	17.453	17.342
SemiC	16.856	16.157
cogproc	–38.140	–38.057
sad	19.972	20.991
affiliation	37.245	37.323
anx	17.692	18.524
death	22.654	23.873
social	83.284	83.246
Analytic	–17.013	–16.901

^a^LIWC: Linguistic Inquiry and Word Count.

Second, we used an Empath-based approach to address the shortcomings of the LIWC-based approach. LIWC can only analyze a total of 6400 dictionary words. A dynamic deep learning–based approach was used to analyze these words with Empath. We analyzed the text with Empath by using the empath-client Python implementation package [43]. Empath draws connotations between words and phrases by using deep learning–based neural embedding across more than 1.8 billion words of modern fiction. Given a small set of seed words that characterize a category, Empath uses its neural embedding to discover new, related terms and then validates the category with a crowd-powered filter. Empath analyzed text across 200 built-in, prevalidated categories we generated from common topics in our data set, like neglect, government, and social media. We analyzed the tweets of each user by using Empath and considered the outcome of the tweets as our independent variables; meanwhile, Insomnia Yes or Insomnia No was taken as the dependent variable. Although LIWC and Empath are highly correlated (r=0.906), we found no correlation between Empath and our insomnia classification categories. LIWC has a total of 93 psycholinguistic categories, whereas Empath has a total of 200 word categories. When these 93 LIWC word categories were divided between 200 Empath word categories, which might not have been evenly distributed across the categories, Empath showed weak correlation coefficients. Therefore, we ultimately did not integrate the Empath word categories with our combined model.

Third, to find correlations of users’ sleeping patterns with the Big 5 personality traits, we initially computed users’ personality scores. We computed the Big 5 personality traits [20] of the users from their tweets by using the IBM Watson Personality Insight API (application programming interface) [44]. Arnoux et al [8] have shown that the IBM Watson Personality Insight API performs well in comparison to other techniques. Other prior studies [45,46] have also used the API and demonstrated reasonable performance. We found that the majority of the traits had a high score of 1.00 and low score of 0.01. We also observed that insomniac users had average openness, conscientiousness, extraversion, agreeableness, and neuroticism scores of 0.61, 0.29, 0.52, 0.56, and 0.83, respectively.

#### Building Classifiers

After feature selection of all the techniques was completed, we first built a classification model with LIWC. To build the classification model, we considered 14 different LIWC categories of words as initial features. Table 3 shows the features for the LIWC-based approach. The features are i, negate, swear, health, drives, focuspresent, SemiC, cogproc, sad, affiliation, anx, death, social, and Analytic. A potential problem can arise when collinearity is present among the features. To remove collinearity among independent LIWC features, we computed the correlation among the features by using the R regression subset selection package “leaps” [47]. We found that the following features were collinear: drives, sad, SemiC, focuspresent, and affiliation. We discarded these collinear features from our feature list. Finally, we conducted classification with the relevant features using a 10-fold cross-validation with 10 iterations. We built our classification model with several classifiers that included Naive Bayes, AdaBoost, random forest, support vector machine, and Gaussian process.

After this, we used a linguistic model that is capable of finding contextual relationships between words in a sentence. To this end, we use the BERT [32] model which is pretrained on a large corpus of sentences. The model learns to produce a powerful internal representation of words as embeddings. We used the sentence-transformers [48] library to generate BERT vectors by using a pretrained model. We grouped 2 sets of preprocessed data sets, one without applying lemmatization and punctuation marks and the other after applying all the preprocessing techniques.

We used the BERT vector as input for our convolutional neural network (CNN)–based deep learning model [49]. As CNN is a nonlinear machine learning model and the BERT embedding vector has a large input feature (768 × 1), we trained our model with this deep learning–based architecture. The CNN model contains 2 hidden layers. In the first hidden layer, we added the leaky ReLU (rectified linear unit) [50] activation function. In the next hidden layer, we added a dropout for regularization of the model with a tanh activation function. Finally, we added a dense layer with a softmax activation function [51] and used the Adam optimizer with binary cross-entropy loss. We split the training and test data sets by 70% and 30%, respectively, and built the classification model using a 10-fold cross-validation with 10 iterations. With this configuration, the accuracy was 67% and 58% for the training and test data sets, respectively.

Next, we built our classification model by using the Big 5 personality traits. Based on the date summarized in Table 4, we selected 3 relevant Big 5 traits: conscientiousness, neuroticism, and agreeableness. We built the classification model with the relevant traits using a 10-fold cross-validation with 10 iterations. We built our classification model with several classifiers that included Naive Bayes, AdaBoost, random forest, support vector machine, and Gaussian process by again splitting the training and test data sets by 70% and 30%, respectively.

**Table 4:** Fisher’s correlation coefficient between personality traits and Insomnia Yes and Insomnia No.

Personality trait	Insomnia Yes (Fisher’s score)	Insomnia No (Fisher’s score)
Conscientiousness	31.885	29.227
Neuroticism	28.168	17.336
Openness	–19.137	–20.785
Extraversion	–1.93	–1.61
Agreeable	2.175	3.132

#### Building Weighted Ensemble Classifiers

We finally combined the previous classification models to increase the strength of our prediction model. To determine a unified final insomnia label, we combined all the independent models, including, LIWC, BERT, and the Big 5 personality traits. It was necessary to prioritize the approaches based on their performance. We ordered the approaches by assigning weights to each model. We used these weights to build our final ensemble model. To build our ensemble model, we performed the following 2 steps: computing weights of each approach from both the training and test data sets and combining the models with a double-weighted linear ensemble technique.

We then determined the weights of each approach (ie, W_LIWC_, W_BERT_, and W_Big5_ personality traits) from both the training and test data sets. We occasionally observed that a model showed better strength with the training data set while the model performed weakly for the test data set. Therefore, we paid close attention to the performance of our models in both the training and test data sets. This way, we could bring greater diversity to the weights to build our ensemble model. To this end, we first ran the classification model over the training data set of 944 Twitter users (944/1574, 60.0% of the total data set). We ran the classification models by using LIWC, BERT, and the Big 5 personality traits. We used both linear (eg, random forest, Naive Bayes) and nonlinear (ie, CNN) models during training. We ranked the strength of these classification models by checking their root mean square error (RMSE) scores. The lower the RMSE score, the higher the weight of a model (linear or nonlinear). To compute the weight, we subtracted the RMSE score from 1 (W=1–RMSE).

**Figure 1:**
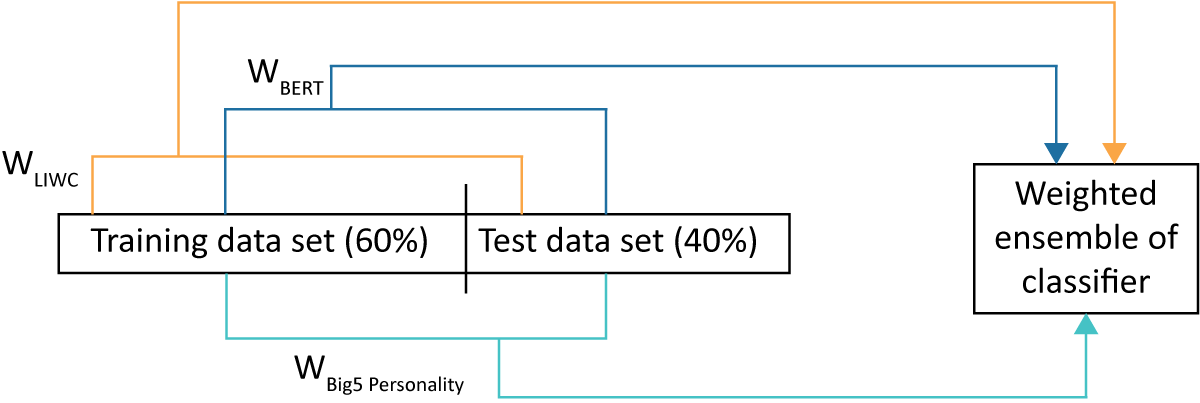
Weight computation and building ensemble model for predicting insomnia. BERT: bidirectional encoder representations from transformers; LIWC: Linguistic Inquiry and Word Count.

We again ran the classification models over the test data set of 630 Twitter users (630/1574, 40.0% of the total data set). We also applied both linear and nonlinear techniques by using LIWC, BERT, and the Big 5 personality trait approaches. We then ranked the weights (1–RMSE for the test data set) of these approaches. Figure 1 shows the detailed process of producing weights from both the training (60%) and test (40%) data sets. In the figure, we indicate weight as W, which indicates that we subtracted RMSE from 1 for the model. In our double-weighted method, we combined the weights of each type by using convex combination techniques [52] for both the training and test data sets. For the training data set, we obtained weights of 0.52, 0.47, and 0.50 from LIWC, BERT, and the Big 5 traits, respectively. In contrast, for the test data, we achieved weights of 0.38, 0.50, and 0.36 from LIWC, BERT, and the Big 5 traits, respectively. These weights were generated from linear and nonlinear classification models of LIWC, BERT, and the Big 5 personality traits.

Finally, we combined the weights that we found from the double-weighted method by using the convex combination method. Equation 1 presents the final insomnia ensemble classification result (*I_Final_*) from the previous 3 different models.

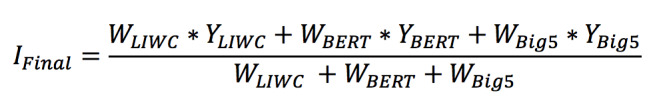



where *Y_LIWC_, Y_BERT_*, and *Y_Big5_* refer to insomnia prediction results through use of LIWC, BERT, and the Big 5 personality traits, respectively; and *W_LIWC_, W_BERT_,* and *W_Big5_* denote the weights generated from LIWC, BERT, and the Big 5 personality traits, respectively, from both the training and test data sets through use of the convex combination technique.

## Results

### Overview

Our study is the first study to build a novel ensemble learning model to predict insomnia by analyzing a large number of tweets. Prior studies [28,29] investigated the pattern of insomniac behavior by using only a limited number of tweets. Furthermore, the authors explicitly considered phrases such as “insomnia” and “sleepless” in their data sets. In contrast, in our study, we discarded these explicit phrases when predicting users’ sleeping issues, which makes our study different and more robust than the previous studies. In this section, we report the performance of our independent and final ensemble-based classifiers, discuss the incorporation of the emotional variability among the insomniac and noninsomniac users from their tweets, and present the distribution of insomniac users and the variability of their Big 5 personality scores. Finally, the correlation between users’ genders and their insomniac behavior is discussed.

### Performance of Independent and Ensemble Classifiers

First, we investigated the performance of our independent and ensemble-based classifiers. Table 5 shows the performance of the classifiers. We found that the Gaussian process classifier had the best average performance (area under the curve [AUC] 75.3%) in predicting users’ insomnia.

**Table 5:** Strength of the classification model for predicting insomnia by LIWC categories and the Big 5 personality traits.

Classifier, insomnia class		LIWC^a^				Big 5 personality traits		
		TPR^b^	FPR^c^	AUC^d^	TPR		FPR	AUC
**Random forest**								
	Yes	0.716	0.334	0.747	0.686		0.475	0.649
	No	0.678	0.270	0.747	0.525		0.314	0.649
**Naive Bayes**								
	Yes	0.740	0.472	0.694	0.746		0.591	0.585
	No	0.536	0.260	0.694	0.409		0.254	0.585
**SVM** ^e^								
	Yes	0.632	0.266	0.680	0.883		0.674	0.604
	No	0.732	0.371	0.680	0.326		0.117	0.604
**AdaBoost**								
	Yes	0.699	0.414	0.694	0.836		0.679	0.599
	No	0.579	0.314	0.694	0.321		0.164	0.599
**Gaussian process**								
	Yes	0.747	0.383	0.754	0.765		0.542	0.666
	No	0.713	0.376	0.754	0.457		0.234	0.666

^a^LIWC: Linguistic Inquiry and Word Count.

^b^TPR: true-positive rate.

^c^FPR: false-positive rate.

^d^AUC: area under the curve.

^e^SVM: support vector machine.

**Figure 2:**
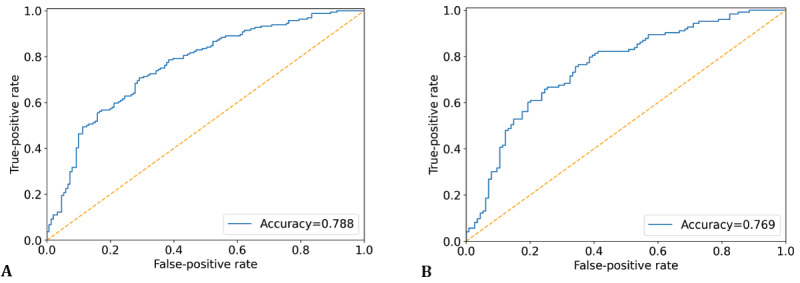
Receiver operating characteristic curves for insomnia classification for the (A) training set and (B) test set.

Our final ensemble classification model for insomnia, *I_Final_*, achieved an AUC of 78.8% and 76.91% from the training and test data sets, respectively, outperforming the previous independent models. When observing the performance of the ensemble-based classifiers on the training and test data sets, we found that the performances in the test set were similar to those of the training data set. The receiver operating characteristic curves for the training and test data sets on our ensemble-based classifiers are displayed in Figure 2.

### Emotional Variability Among Insomniac and Noninsomniac Users

**Figure 3:**
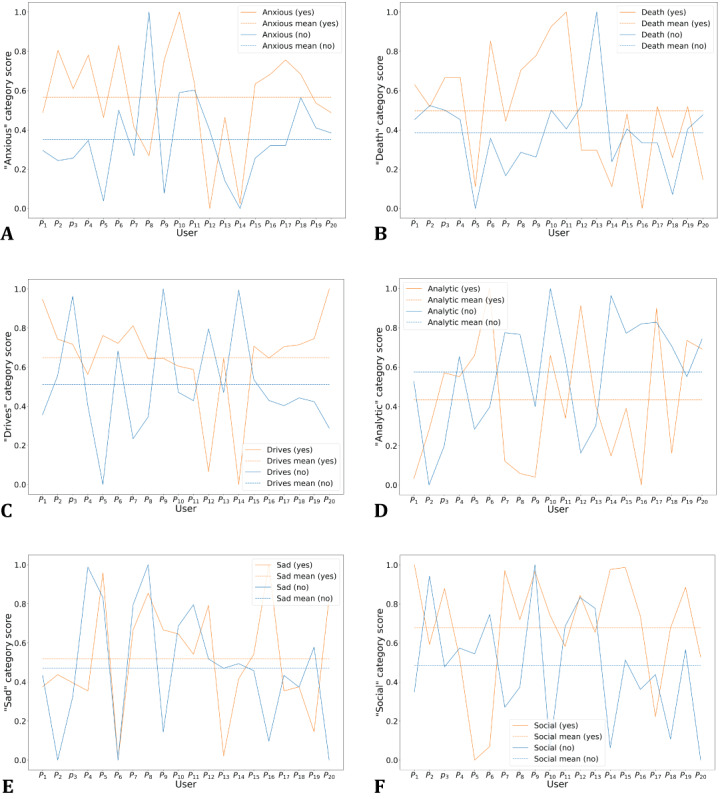
Usage of words, (A) “Anxious,” (B) “Death,” (C) “Drives,” (D) “Analytic,” (E) “Sad,” and (F) “Social,” related to the LIWC category and their mean scores between insomniac and noninsomniac users. LIWC: Linguistic Inquiry and Word Count.

Our study investigated whether emotional variability exists between insomniac and noninsomniac users based on their tweets. From our observation, we found that users’ insomniac behavior and their psycholinguistic categories were correlated. We randomly selected 20 insomniac and 20 noninsomniac users. We extracted users’ anxiety-related words, (eg, “worried,” “fearful,” “nervous,” “tense”) and converted their scores into a range from 0 to 1 by using the max-min normalization technique. Figure 3 presents the variability in the use of these words between the insomniac and noninsomniac users. We also observed that on average, insomniac users exploited anxiety-related words 10% more than did the noninsomniac users. Carrera et al [53] showed in their study of 200 college students that a significant association can be found between difficulty sleeping and fear of death. In our study, we also found that insomniac users are likely to post death-related words in their tweets. We observed that insomniac users write more “social words.” These users tend to spend more time socializing with others when they face difficulty sleeping. Our study also showed that insomniac users tend to display a lack of analytical thinking.

### Visualization of the Correlation Between Big 5 Personality Traits and Insomniac Users

Users’ Big 5 personality traits and insomniac behavior showed correlations. Table 6 shows the distributions indicating that agreeableness might have a weak correlation with insomniac behavior. However, conscientiousness had a strong correlation with insomniac behavior (Fisher’s score 31.88). The users who had high insomnia were more likely to be more conscientious. In contrast, Wissar et al [54] reported that lower conscientiousness is associated with greater insomnia severity. We also find that users who had high neuroticism scores were more likely to have severe insomnia. Table 6 shows that insomniac users have moderate scores in agreeableness, are likely to be high in conscientiousness, and are highly neurotic.

**Table 6:** The distribution of correlated Big 5 personality trait scores among insomniac users.

Traits by range		Percentage, n (%) (N=820)	
**Agreeableness**			
	0.0–0.2		77 (9.4)
	0.2–0.4		164 (20.0)
	0.4–0.6		188 (22.9)
	0.6–0.8		214 (26.1)
	0.8–1.0		177 (21.6)
**Conscientiousness**			
	0.0–0.2		111 (13.5)
	0.2–0.4		213 (26.0)
	0.4–0.6		376 (45.9)
	0.6–0.8		88 (10.7)
	0.8–1.0		32 (3.9)
**Neuroticism**			
	0.0–0.2		9 (1.1)
	0.2–0.4		38 (4.6)
	0.4–0.6		79 (9.6)
	0.6–0.8		125 (15.2)
	0.8–1.0		569 (69.4)

### Insomnia and Gender Correlation

Seabrook et al [39] showed that gender might have an association with mental health problems, such as depression. Reasoning that insomnia is a mental health problem [55] and motivated by previous studies [39,56], we also investigated the association between users’ gender and their insomnia-related problems. We discovered that the number of insomniac and noninsomniac male users was 309 and 363, respectively. We further observed that the number of insomniac and noninsomniac female users was 511 and 391, respectively. From our data set, we conducted chi-square (*P*<.001) tests based on gender and insomnia where the degrees of freedom ([number of rows–1] × [number of columns–1]) in the contingency table were 1. Our result showed that gender and insomnia were correlated. 

After careful observation, we found that among 511 of all female users 6.8% (n=35 users) were suffering from pregnancy- or postpartum-induced insomnia. These users’ tweets contain words related to “pregnant,” “postpartum,” and “cosleep.” Later, we applied LDA [57] over the tweets from these users’ pregnancy or postpartum times. LDA is a topic modeling technique that automatically organizes a large corpus to discover hidden themes. From users’ patterns of tweeting, we could estimate the possible time frame for their pregnancies. Table 7 presents the extracted 5 major topics from their tweets: sleep, night, tired, child, and weird.

**Table 7:** Distribution of major topics, including (A) sleep, (B) child, (C) night, (D) tired, and (E) weird, extracted from a group of insomniac users’ tweets during their pregnancy or postpartum periods.

Topic		Distribution (%)
**Topic A**		
	Sleep	0.085
	Problem	0.077
	Suffer	0.075
	Little	0.045
	Anxiety	0.060
	Dream	0.057
	Escape	0.037
	Spiritual	0.036
	Random	0.035
	Morning	0.030
**Topic B**		
	Birth	0.081
	Breast	0.071
	Nursing	0.070
	Happiness	0.076
	Lucky	0.062
	Potty	0.079
	Scare	0.050
	Check	0.048
	Patience	0.052
	Angel	0.058
**Topic C**		
	Tonight	0.072
	Night	0.070
	Dinner	0.061
	Noise	0.060
	Think	0.072
	Party	0.048
	Event	0.051
	Pizza	0.042
	Worry	0.050
	Carry	0.049
**Topic D**		
	Tired	0.076
	Suffer	0.068
	Watch	0.061
	Stressful	0.060
	Minute	0.059
	Break	0.070
	Hyperemesis	0.043
	Pregnancy	0.042
	Battle	0.040
	Sport	0.039
**Topic E**		
	Weird	0.074
	Stare	0.062
	Disgust	0.060
	Crazy	0.056
	Bitch	0.072
	Shame	0.050
	People	0.070
	Employee	0.049
	Dislike	0.069
	Pregnant	0.068

## Discussion

### Emotional Linguistic and Insomniac Behavior

As discussed earlier, users’ linguistic patterns and Insomnia Yes or Insomnia No indications were strongly correlated. Insomniacs tended to use negative categories of LIWC words (eg, “no,” “not,” “never”). Some people with strong temperament frequently use swear words (eg, “damn,” “piss”). In Bonnet et al’s [55] study, insomniacs showed hyperarousal (an abnormal state of responsiveness) due to increased secretion of corticosteroids and adrenaline and an elevated metabolic rate. In another study, Bonnet et al [58] showed that insomniacs experience mood alternation and chronic psychological activation. The following is a sample tweet from an Insomnia Yes user that endorses our finding: “It sucks when you realize that the people closest to you are the most toxic.” They also use anxious (eg, worried, fearful, nervous) and sad (eg, crying, grief, sad) categories of LIWC words in their tweets. Freeman et al [59] showed that insomnia has a connection with anxiety, depression, and being worried, and these people are likely to use death-related words (eg, “bury,” “coffin,” “kill”). Harrison et al [60] reported that insomniacs generally exhibit fear of death and show anxiety about the uncertain (eg, “maybe,” “perhaps,” “guess”). The following tweet is an example this: “I feel dead and I hate everybody.” Our study also adds to these findings by analyzing the correlation between LIWC categories of words found in the users’ tweets and their sleeping patterns. Hiller et al [61] showed that cognitive processes (eg, the psychobiological inhibition model) play an important role in understanding and treating insomnia. We obtained a few correlations, such as focuspresent and SemiC, which cannot be intuitively explained.

We did not find a correlation between Empath categories of words and users’ sleeping patterns. It is interesting to observe that none of these categories showed any significant correlation between Empath word categories and users’ sleeping habits. Fast et al [62] reported that LIWC and Empath word categories are correlated (r=0.906). LIWC has a total of 93 word categories that are distributed across a total of 200 word categories in Empath. When a word category subsumes a greater number of individual words, then the chance of 2 features being correlated, such as LIWC and insomnia class labels, increases. In contrast, when the number of words decreases in a category, such as in Empath, then the possibility of being correlated decreases. In our study, we observed that although LIWC and Empath were correlated, this correlation might not have been sustained in a transitive case. For example, although Empath → LIWC and LIWC → Feature_x_ are true, Empath → Feature_x_ does not exist in our data set.

### The Big 5 Personality Traits and Insomniac Behavior

We discovered that users’ personalities and their sleeping patterns have strong correlations and that users with high neuroticism are more likely to have insomnia. They tend to go through depression, social introversion, repression, and intolerance in terms of somatic health problems. The following is a sample tweet supporting this finding: “Ralph Northam needs to fucking RESIGN already. I have zero tolerance for him.” A prior study [63] also reported similar observations related to the correlation between neuroticism and insomnia. Assessing neuroticism may allow for the early detection of catastrophic situations of insomnia. We also observed that users with high conscientiousness scores had strong correlations with insomniac patterns. The following tweet from our data set exemplifies this finding: “I cannot control others actions but I can control mine and my reactions.” Larsgaard et al [64] noticed that conscientiousness in people might be correlated with reduced sleeping behavior due to their self-inhibition and the meticulousness in their daily activities.

In our study, we also found a correlation, albeit a weak one, between users’ agreeableness and insomnia. The following tweet from an Insomnia Yes user is an example of this: “understand people who talk crap about others thru social media. Listen Linda, why aren’t you handling it like an adult?” Dekker et al [65] found that neuroticism, agreeableness, and openness are directly associated with the insomnia severity index. In our study, we discovered that openness is negatively correlated with users’ sleeping patterns. Tsaousis et al [66] also demonstrated that openness and insomnia are negatively correlated with each other. However, several studies [67-70] have demonstrated that openness is unrelated to sleep quality.

In our study, there was negative correlation between extraversion and insomnia. Gray et al [69] found there to be no correlation between users’ extraversion and insomniac patterns. Our observations of the relationship between the Big 5 traits and insomnia largely overlap with prior findings [63-65,67-70], which suggest that investigating users’ insomnia patterns through social media can leverage our manual effort without asking an individual directly about their sleeping issue. Furthermore, understanding and inspecting one’s personality traits may provide clues to underlying causes of vulnerability to developing insomnia.

### Pregnancy and Insomniac Behavior

According to our finding, users share their depression during their pregnancies and share their experiences during their postpartum periods. We observed that the major supporting topics were night, suffer, problem, and birth, among others. We also found a few topics that described parents’ postpartum-related anxiety. Important topics that we found from users’ tweets were birth, breast, tired, suffer, and random, among others. Previous studies also support our findings [71,72].

### Conclusions

In this study, we investigated the associations between the psycholinguistic and personality traits of users’ with insomnia. We captured both the word usage and semantics of users’ tweets through the LIWC and BERT linguistic models and developed 2 models to predict insomnia from LIWC features and BERT word embedding. We then built our third machine learning model that used derived personality traits to predict insomnia from tweets. Finally, we built a rigorous ensemble model by combining the 3 separate models. Our ensemble classifier showed strong prediction potential (AUC 78.8%). The classifier was built by using a novel, double-weighted ensemble technique that outperformed the independent classifiers. We plan to improve our classifier by integrating more data from social networks, such as friend lists, time of tweets, gender, workplace, time spent on activities, etc. We also plan to analyze tweets in different languages.

## Abbreviations

API: application programming interface
AUC: area under the curve
BERT: bidirectional encoder representations from transformers
CNN: convolutional neural network
LDA: latent Dirichlet allocation
LIWC: Linguistic Inquiry and Word Count
ReLU: rectified linear unit
RMSE: root mean square error
